# Divergent DNA methylation patterns and gene expression in MYC and *CDKN2B* in canine transmissible venereal tumors

**DOI:** 10.14202/vetworld.2024.1581-1590

**Published:** 2024-07-24

**Authors:** Soukkangna Keopaseuth, Kidsadagon Pringproa, Prapas Patchanee, Chanokchon Setthawongsin, Somporn Techangamsuwan, Phongsakorn Chuammitri

**Affiliations:** 1 Graduate Program in Veterinary Medicine, Faculty of Veterinary Medicine, Chiang Mai University, Chiang Mai, 50100 Thailand; 2 Veterinary Bioscience Unit, Veterinary Academic Office, Faculty of Veterinary Medicine, Chiang Mai University, Chiang Mai, 50100 Thailand; 3 Veterinary Academic Office, Faculty of Veterinary Medicine, Chiang Mai University, Chiang Mai, 50100 Thailand; 4 Department of Veterinary Nursing, Faculty of Veterinary Technology, Kasetsart University, Bangkok 10900, Thailand; 5 Center of Excellence for Companion Animal Cancer, Department of Pathology, Faculty of Veterinary Science, Chulalongkorn University, Bangkok, 10330 Thailand

**Keywords:** canine transmissible venereal tumor, *CDKN2B*, DNA methylation, MYC, oncogene, tumor suppressor gene

## Abstract

**Background and Aim:**

Canine transmissible venereal tumor (CTVT), a unique transmissible cancer in dogs, affects the external genitalia and potentially spreads to other parts of the body. While somatic mutations in oncogenic and tumor-suppressing genes are linked to CTVT development, the impact of DNA methylation, which affects gene expression, remains unclear. This study explored whether DNA methylation in the promoter regions of the MYC oncogene and *CDKN2B* tumor suppressor genes in CTVTs is associated with their expression, both at the gene and protein levels.

**Materials and Methods:**

To investigate promoter DNA methylation of MYC and *CDKN2B* in CTVTs, we analyzed frozen tissue samples from genital CTVT (GTVTs) and extragenital CTVT (ETVTs). Genomic DNA was extracted, bisulfite-treated, and analyzed using bisulfite polymerase chain reaction (PCR) and sequencing. The messenger RNA and protein of MYC and *CDKN2B* were also extracted and assessed by real-time PCR and Western blotting. Matching formalin-fixed, paraffin-embedded blocks were used for immunohistochemical staining to visualize protein distribution in GTVT and ETVT tissues.

**Results:**

Although both GTVT and ETVT samples showed MYC promoter methylation, the extent of methylation differed significantly. GTVTs displayed a much higher degree of methylation, potentially explaining the more pronounced downregulation of MYC gene expression and reduction in c-MYC protein levels observed in GTVTs compared with ETVTs. Our data revealed a prevalent hypermethylation pattern in the *CDKN2B* promoter across both sample types. However, DNA methylation, which was expected to have a suppressive effect, did not correlate with gene/protein expression. GTVTs displayed high protein levels despite significantly reduced *CDKN2B* expression. Conversely, ETVTs maintained regular *CDKN2B* expression but exhibited reduced protein production, suggesting a complex interplay between methylation and expression in these tumors.

**Conclusion:**

MYC demonstrated a clear association between its promoter methylation status, gene expression, and protein levels; however, *CDKN2B* lacked this correlation, implying the involvement of methylation-independent regulatory mechanisms and highlighting the need for further investigation.

## Introduction

Canine transmissible venereal tumor (CTVT) and devil facial tumor disease 1 (DFT1), previously referred to as devil facial tumor disease, are two well-known clonally transmissible cancers in terrestrial animals [1, 2]. CTVT, a unique cancer, can spread through damaged skin and mucosa in immune-compromised animals through viable cancer cells, acting as both an allograft and xenograft [3−5]. Through a process known as CTVT metastasis, this tumor can migrate to other parts of the body, including the nasal cavity, eyes, oral cavity, skin, and lymph nodes [3, 6]. CTVT development is characterized by a plethora of genetic abnormalities, including somatic mutations, deletions, duplications, inversions, translocations, and genetic instability. This complex genetic landscape, resulting in an aneuploid genome, empowers tumor cells to reprogram their gene expression, promoting uncontrolled growth and self-renewal through telomerase activity [5, 7, 8]. The CTVT genome comprises chromosomal aberrations, loss of heterozygosity, and various distinct mutations, such as alleles inherited from the ancestral lineage and lineage-specific somatic mutations [9−11]. Frequent gene modifications involve a combination of proto-oncogenes, tumor suppressor genes (TSGs), apoptosis-associated genes, and methylation processes. Researchers have focused on identifying early driver gene mutations in the proto-oncogene MYC and the TSGs *CDKN2A* and *CDKN2B* because these genes are often mutated in CTVT, and mutations in these genes are distinctive and essential for CTVT survival [1, 2, 7, 12].

DNA methylation plays a pivotal role in cancer genesis, progression, regression, gene expression, and genomic integrity, and it is altered in cancer, with global hypomethylation leading to oncogene reactivation and promoter hypermethylation leading to TSG silencing [4, 13]. DNA methylation silences genes by preventing transcription factors (e.g., activator protein-2 and nuclear factor-kappa B) from binding to their DNA sequences and recruiting proteins that repress transcription. This occurs most often in CpG island (CGI) shores and exons near the 3′ end of a gene [14, 15]. Hypomethylation can lead to oncogene activation (e.g., c-MYC and H-RAS75), whereas hypermethylation can silence TSGs and cell cycle control genes (e.g., CDKN2A, CDKN2B, and Rb). This can affect cancer stem cells and the phenotype of CGIs [14].

The information above applies to CTVT and transmissible venereal tumor (TVT) in Tasmanian devils, with evidence suggesting epigenetics and DNA methylation’s involvement in DFT1 evolution, TVT malignancy development, and its changes during CTVT regression [4, 16, 17]. Although DNA methylation analysis of oncogene and TSG promoters is a common clinical technique, a consensus on the specific methylation status of genes crucial to canine TVT remains elusive. This study explored how DNA methylation in CTVT gene promoters, particularly MYC (oncogenic) and *CDKN2B* (tumor suppressor), affects gene and protein expression.

## Materials and Methods

### Ethical approval

The tumor biopsy samples came from dogs diagnosed with CTVT at the Oncology Unit of the Small Animal Teaching Hospital, Faculty of Veterinary Science, Chulalongkorn University, Thailand. All procedures were performed under local anesthesia with approval from the Chulalongkorn University Animal Care and Use Committee (approval number 133100077).

### Study period and location

The data collection for this study was conducted from April 2022 to May 2023 at the Center of Veterinary Medical Diagnostic and Animal Health Innovation, Chiang Mai University, Thailand.

### CTVTs tissue samples

This study used 10 samples of CTVT tissue from dogs with clinical manifestations. The samples included six frozen tissue samples from genital CTVTs (GTVTs) and four from extragenital CTVTs (ETVTs) in the skin, oral, or nasal cavity. Samples were obtained from the Oncology Unit of the Small Animal Teaching Hospital at the Faculty of Veterinary Science, Chulalongkorn University, Thailand. These samples were subsequently transferred to the Center of Veterinary Medical Diagnostic and Animal Health Innovation at Chiang Mai University, Thailand, for further processing and analysis. The origins of these samples can be found in previously published studies [18, 19]. Blinding was not used in this study. In addition to frozen tissues, we used six available formalin-fixed, paraffin-embedded (FFPE) blocks (n = 3 each of GTVTs/ETVTs) that matched the previously described samples.

### Genomic DNA (gDNA) extraction and bisulfite-modified gDNA (bisulfite modification)

Frozen tumor tissues (100 mg) were used for gDNA extraction using DNAzol® (MRC, Cincinnati, OH, USA) according to the manufacturer’s recommendations. Following DNA extraction, the gDNA was subjected to bisulfite treatment to enable subsequent methylation analysis. In brief, 500 ng of gDNA was bisulfite modified using the EZ DNA Methylation-Gold™ kit (Zymo Research, Irvine, CA, USA). Bisulfite-treated DNA (bsDNA, 100 ng/μL) was stored at −20°C until further use.

### Bioinformatics and primer information

**Figure-1:**
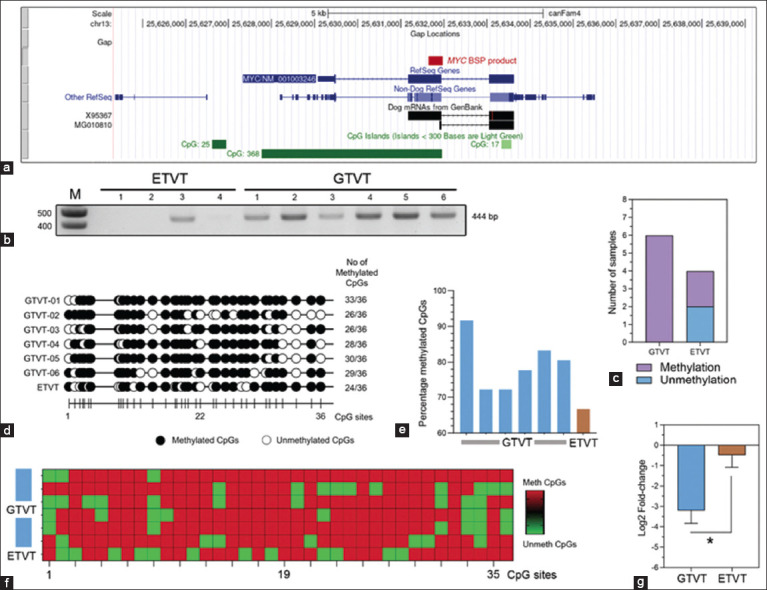
BSP analysis for MYC promoter methylation and MYC expression by real-time PCR in GTVTs and ETVTs, (a) Genomic coordinate data of the canine MYC gene displayed using the University of California, Santa Cruz (USCS) genome browser. The red box indicates the BSP product of MYC in this study, whereas the green boxes indicate the CGIs at the promoter region of the canine MYC gene. (b) Representative agarose gel electrophoresis of the BSP products from amplification of the MYC gene from GTVTs (n = 6) and ETVTs (n = 4). The correct PCR product with a size of 444 bp was detected in all samples, indicating the presence of methylation in the PCR amplicons. The leftmost lane shows a DNA marker. (c) Stacked bar graph shows the percentage of MYC genes that are methylated and unmethylated in GTVTs and ETVTs. The data for this graph were derived from (b). (d) A lollipop methylation diagram shows the methylation status of CpG sites in the MYC PCR products from GTVTs (n = 6) and ETVT (n = 1). The number of methylated CpG sites is shown at the end of the diagram. The nucleotide position of each CpG site is depicted at the bottom of the lollipop. Black circles represent the methylated sites, whereas the white circles indicate the unmethylated positions. The diagram was created using QUMA online software. (e) Bar graphs show the percentage of methylation of the canine MYC gene promoter region in GTVTs (n = 6) and ETVT (n = 1). (f) Heat map shows the methylation status of each position in the MYC promoter sequence. (g) Bar graphs show the relative expression of MYC messenger RNA (log2 fold-change) in GTVTs (n = 6) and ETVTs (n = 4). Data are presented as mean ± standard error of the mean and statistical analysis was performed by unpaired t-test. *p < 0.05 between groups. BSP=Bisulfite sequence polymerase chain reaction, PCR=Polymerase chain reaction, GTVT=Genital canine transmissible venereal tumors, ETVT=Extra-genital transmissible venereal tumors.

**Figure-2:**
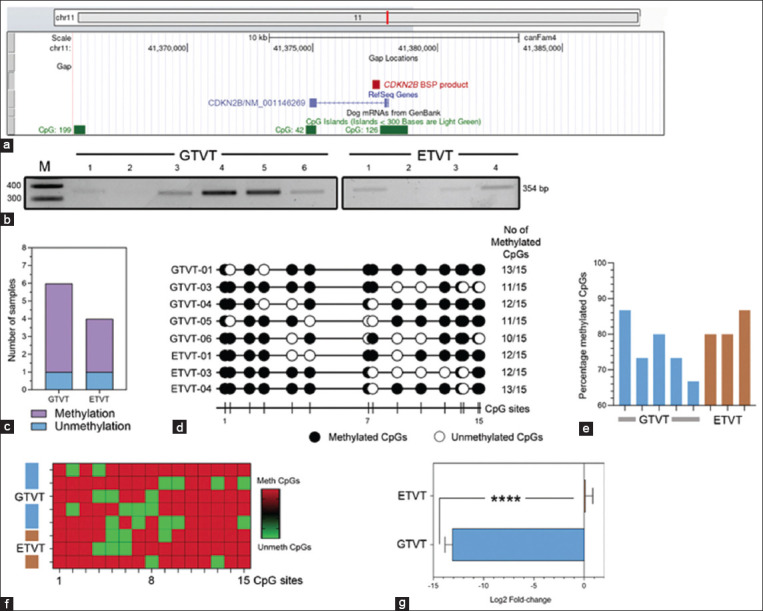
BSP analysis for *CDKN2B* promoter methylation and *CDKN2B* expression by real-time PCR in GTVT and ETVT, (a) Genomic coordinate data of the canine *CDKN2B* gene displayed using the USCS genome browser. (b) Representative agarose gel electrophoresis of the BSP products from amplification of the *CDKN2B* gene (354 bp) from GTVTs (n = 6) and ETVTs (n = 4). (c) Stacked bar graph shows the percentage of *CDKN2B* genes that are methylated and unmethylated in GTVTs and ETVTs. The data for this graph were derived from (b). (d) A lollipop methylation diagram shows the methylation status of CpG sites in the *CDKN2B* PCR products from GTVTs (n = 5) and ETVTs (n = 3). Black circles represent the methylated sites, whereas the white circles indicate the unmethylated positions. (e) Bar graphs show the percentage of methylation of the canine *CDKN2B* gene promoter region in GTVTs (n = 5) and ETVTs (n = 3). (f) Heat map shows the methylation status of each position in the *CDKN2B* promoter sequence. (g) Bar graphs show the relative expression of *CDKN2B* messenger RNA (log2 fold-change) in GTVTs (n = 6) and ETVTs (n = 4). Data are presented as mean ± standard error of the mean and statistical analysis was performed by unpaired t-test. ****p < 0.0001 between groups. BSP=Bisulfite sequence polymerase chain reaction, PCR=Polymerase chain reaction, GTVT=Genital canine transmissible venereal tumors, ETVT=Extra-genital transmissible venereal tumors.

The search for a CGI in the promoter region of MYC and *CDKN2B* genes was performed using the University of California, Santa Cruz (UCSC) Genome Browser, assembly ID: canFam3 (Figures-1a and 2a). The primer sequences used for bisulfite sequence polymerase chain reaction (BSP) at the promoters of genes: Canine MYC and *CDKN2B* are indicated in Table-1 [20, 21]. The messenger RNA (mRNA) expression of the genes involved in CTVTs: MYC and *CDKN2B*, and the reference gene (*GAPDH*), are indicated in Table-1.

**Table-1:** List of primer sequences and related information used in this study.

Gene	Primer	Sequence	Size (bp)	Annealing Temp (°C)	Reference
Bisulfite PCR					
MYC	Forward	GGAGAAGTTGGTTTTTTATTAG	444	55	[20]
	Reverse	TTTCCCTTCCTAAAACTAAAA			
*CDKN2B (p15)*	Forward	GTGAGGTTGTGGGGTTTAG	354	59	[20]
	Reverse	AACCTCCCAATACAAATAATTCA
Real-time PCR					
MYC	Forward	TGAAACGGAGCTTCTTTGCC	156	60	This study
	Reverse	TCCGCAACAAGTCCTTTTCG			
*CDKN2B*	Forward	GCGGCAGCTCCTGGAAG	125	60	[21]
	Reverse	GGGTCGGCACAGTTGG			
*GAPDH*	Forward	TCACCAGGGCTGCTTTTAAC	129	60	This study
	Reverse	TGACTGTGCCGTGGAATTTG			

PCR=Polymerase chain reaction

### DNA methylation status of bsDNAs using BSP

BSP was used to evaluate the DNA methylation statuses at the canine MYC and *CDKN2B* promoters in bsDNA from GTVTs and ETVTs. BSP was used to detect methylated amplicons. Polymerase chain reaction (PCR) was performed in 25 μL reaction mixtures containing 2× MyTaq HS red mix (Bioline, Swedesboro, NJ, USA), followed by 43 cycles of PCR. PCR products were then separated on a 1.5% agarose gel and visualized by ethidium bromide staining.

### Purification of amplified PCR products, DNA sequencing, and DNA methylation pattern identification of CGI

The amplified PCR products were electrophoresed, excised, and gel-purified using the HiYield Gel/PCR fragments extraction kit (RBC Bioscience, New Taipei City, Taiwan). Purified PCR products were submitted for direct sequencing at Macrogen, South Korea. The sequences were analyzed using Geneious 8.1.6 (Biomatters, Auckland, New Zealand). The comparative percentage of methylated CpG and unmethylated CpG in the bisulfite sequence data were quantified using QUMA, which is available at http://quma.cdb.riken.jp/.

### RNA extraction and complementary DNA (cDNA) synthesis

Total RNA was extracted from 10 CTVT frozen tissue samples (100 mg each) using RNAzol RT solution (Sigma-Aldrich, St Louis, MO, USA) with a protocol from the manufacturer. cDNA was reverse transcribed from 2 μg of total RNA using the Tetro cDNA synthesis kit (Bioline).

### Real-time quantitative reverse-transcription PCR (qRT-PCR)

Real-time qRT-PCR was performed to investigate gene expression. The primers used in this study are shown in Table-1. Quantification was performed in triplicate on an ABI 7300 real-time PCR system (Life Technologies, Waltham, MA, USA). qRT-PCR was performed using the SensiFAST SYBR Hi-ROX kit (Bioline) according to the manufacturer’s instructions for 40 cycles with an annealing temperature set at 60°C for every tested gene. The relative gene expression level (RQ) was normalized to that of *GAPDH*. The expression levels (log2 fold-change) were determined using the 2^−ΔCt^ method.

### Histology and immunohistochemistry (IHC) on FFPE tissue blocks

For histopathological examinations, tumor tissue samples were dissected, FFPE, cut, and routinely stained with hematoxylin and eosin stain (H&E). The tumor samples were prepared as histopathological slides for IHC assays. The archival FFPE blocks of CTVTs (n = 3 each for GTVTs and ETVTs) were processed and incubated with primary antibodies specific for c-MYC and CDKN2B. The primary antibodies were mouse monoclonal anti-human c-MYC antibody (Clone 9E10, BioLegend, San Diego, CA, USA) and mouse anti-human p15INK4b/CDKN2B (Clone 651308, Novus Biologicals) for 2 h at 25°C. After washing, slides were incubated with horseradish peroxidase (HRP)-conjugated goat anti-mouse polyclonal immunoglobulin G (IgG) antibody (Clone Ply4053, BioLegend) for 1 h. Then, they were incubated with 3,3′-diaminobenzidine tetrahydrochloride (DAB) (Sigma-Aldrich) and immediately washed with tap water after the color developed. The slides were then counterstained with hematoxylin and mounted. For the control of non-specific binding, the primary antibody was replaced with mouse sera. The immunostaining slides were visualized and scanned using a Pannoramic MIDI Slide Scanner (3D HISTECH, Budapest, Hungary). Positive staining of the tissues was evident from the brown.

**Figure-3:**
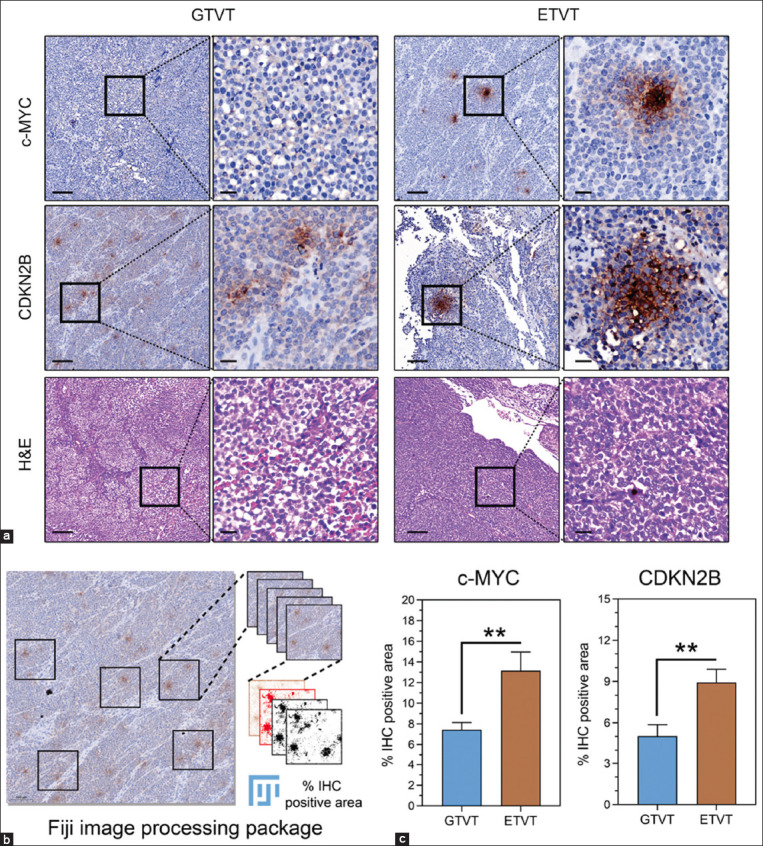
IHC results of c-MYC and CDKN2B in GTVT and ETVT (a) Representative IHC and corresponding H&E images for immunostaining of c-MYC and CDKN2B in canine GTVTs (n = 3) and ETVTs (n = 3). In each section, the left panel depicts the IHC image in low magnification (10×), and the region of the black box (right panel) is enlarged and shown as a high magnification (63×), accompanied by H&E staining results at the bottom. Scale bar, 100 μm and 20 μm for 10× and 63×, respectively. (b) Quantification of IHC images using Image J (Fiji). The five selected areas in each image were converted to 8-bit single-color images using the HDAB color vector. Then, an adjusted threshold was applied to create binary images, where red pixels indicate selected areas. Next, the watershed function is applied to the image to segment the positive IHC areas. Then, the analyzed particles plugin is used to count the percentage of positive IHC areas. (c) Bar graphs show the percentage of IHC-positive are of c-MYC and CDKN2B in GTVTs (n = 15) and ETVTs (n = 15). Data are presented as mean ± standard error of the mean and statistical analysis was performed by unpaired t-test. **p < 0.01 between groups. IHC=Immunohistochemistry, H&E=Hematoxylin and eosin, GTVT=Genital canine transmissible venereal tumors, ETVT=Extra-genital transmissible venereal tumors.

Two independent pathologists randomly selected five areas of each stained IHC slide at 10× magnification, each 325 × 325 pixels in size, with a preference for areas with positive staining signals. Sixty images, including 15 from GTVT and ETVT, were semi-quantitatively analyzed for c-MYC and CDKN2B using Fiji ImageJ (https://github.com/imagej) to quantify deconvoluted DAB images [22]. The percentage of ihc-positives patients between the GTVT and ETVT groups are shown in (Figure-3b).

### Western blot

Total protein from frozen GTVTs (n = 4) and ETVTs (n = 3) was extracted using radioimmunoprecipitation assay lysis buffer (Sigma) supplemented with a protease inhibitor cocktail (Sigma). Protein concentrations were measured using a Bradford protein assay (Bio-Rad, Hercules, CA, USA). Equal amounts (30 μg) of protein were separated by 12%–15% sodium dodecyl-sulfate polyacrylamide gel electrophoresis. The gels were transferred to a 0.2 μm polyvinylidene fluoride or polyvinylidene difluoride (Bio-Rad), followed by blocking in 5% bovine serum albumin (Bio Basic, Markham ON, Canada). The membranes were incubated with mouse monoclonal antibodies for c-MYC (1:1,000), p15INK4b/CDKN2B (1:1,000), and β-actin (1:3,000 as loading control) for 2 h at 25°C with gentle agitation. Next, the membranes were incubated with HRP-conjugated goat anti-mouse IgG antibody (clone 175 Poly4053, BioLegend) at 1:6,000 dilution for 45 min at 25°C. Following membrane washing with tris buffered saline with tween 20, the Opti-4CN substrate kit (Bio-Rad) was used for colorimetric detection. Targeted protein levels were quantified using Image Studio Lite software (LI-COR, Lincoln, Nebraska, USA).

### Statistical analysis

Descriptive statistics were used in the statistical reports to show the number of BSP positives and the percentage of methylated/unmethylated CpG sites. Unpaired t-tests available in GraphPad Prism 9 (GraphPad Software, San Diego, CA, USA) and R version 4.2.1 (R Core Team, https://www.r-project.org/) were used to analyze real-time PCR, IHC, and western blot data. A p < 0.05 indicated statistically significant differences. All data are presented as mean ± standard error. The bar graphs and heatmap were generated using GraphPad Prism.

## Results

### MYC DNA methylation pattern within the CGI and MYC gene expression

BSP was used to detect MYC methylation in GTVT and ETVT samples from dogs with CTVTs (Figures-1a–c). BSP of a 444-bp fragment of the MYC gene promoter showed that all six GTVT samples (6/6) were methylated, whereas only one ETVT sample (1/4) was methylated (Figures-1b and c). Bisulfite genomic sequencing showed that the methylation proportion of the MYC gene varied from 72.22% to 91.67% in GTVTs and 66.67% in ETVTs, as shown in Figures-1d–f. DNA methylation at the promoter region can alter gene expression or contribute to gene silencing. Thus, we investigated the mRNA expression of the oncogenic MYC gene in CTVTs. Real-time PCR (Figure-1g) showed that MYC was downregulated in both GTVs and ETVTs. The expression level in GTVTs (−3.20 ± 0.63) was significantly lower than that in ETVTs (−0.48 ± 0.59; p = 0.018, Figure-1g).

### Methylation pattern of *CDKN2B* adjacent to CGIs and *CDKN2B* gene expression

The *CDKN2B* DNA methylation pattern flanking the CGI was discerned by BSP and DNA sequencing (Figure-2a). Ten samples were tested for *CDKN2B* gene methylation, including six samples of GTVTs and four of ETVTs. BSP of a 354-bp fragment for the *CDKN2B* methylation gene revealed that 5 of 6 GTVT samples and 3 of 4 ETVT samples were methylated (Figures-2b and c). The bisulfite genomic sequencing results displayed methylation proportions of the *CDKN2B* gene spanning from 66.67% to 86.67% in GTVTs and from 80% to 86.67% in ETVTs, as illustrated in Figures-2d–f.

The expression pattern of *CDKN2B* was very similar to that of MYC, but the expression levels were not the same. A notable discrepancy was observed in the mean *CDKN2B* gene expression levels between GTVTs (−13.12 ± 0.72) and ETVTs (0.12 ± 0.71, p < 0.0001; Figure-2g). This suggests the methylation findings from BSP.

### IHC for c-MYC and CDKN2B in canine TVT samples

IHC was applied to analyze the protein, whereas BSP was employed to determine the DNA methylation status of the target genes. We used antibodies against c-MYC and CDKN2B in an IHC study to assess the distribution and localization of these proteins within canine TVTs. The IHC study of c-MYC and CDKN2B in canine TVTs found that most of the staining was focal and confined to the cytoplasm of tumor cells (Figure-3a). ETVT had a significantly higher percentage of c-MYC IHC staining (13.15% ± 1.81%) than GTVT (7.44% ± 0.68%), with a p = 0.0063. Similarly, the percentage area of positive CDKN2B IHC staining was significantly higher in ETVT (8.93% ± 0.97%) than in GTVT (5.03% ± 0.84%), with a p = 0.0049 (Figure-3c). The c-MYC- and CDKN2B-stained tissues showed a narrow range of weak immunostaining intensity and distribution.

### Immunoblotting of c-MYC and CDKN2B protein expression

**Figure-4:**
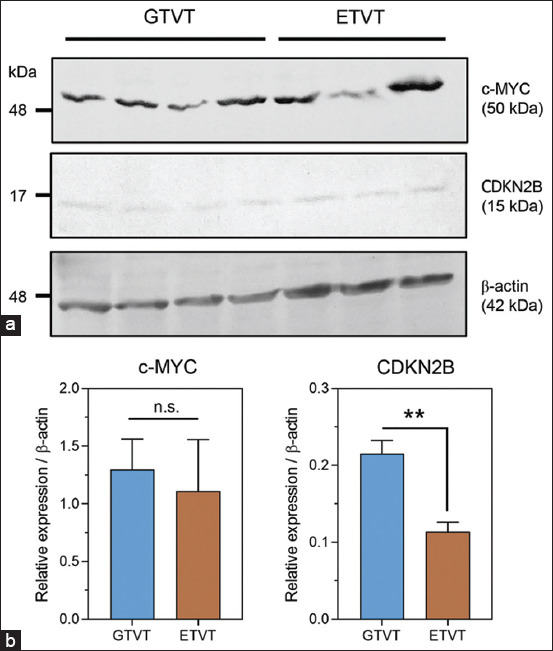
Western blot analysis of c-MYC and CDKN2B in GTVTs and ETVTs (a) An immunoblot of c-MYC (top panel) and *CDKN2B* (middle panel) in tissue lysates from GTVTs and ETVTs is shown, with β-actin (bottom panel) as the loading control. (b) The relative protein expression levels of c-MYC and *CDKN2B* in each tumor tissue are shown in bar graphs, with four samples in the GTVT group and three samples in the ETVT group. Data are presented as mean ± standard error of the mean and statistical analysis was performed by unpaired t-test. **p < 0.01 between groups, n.s=not significant. GTVT=Genital canine transmissible venereal tumors, ETVT=Extra-genital transmissible venereal tumors.

This study used immunoblotting to measure the levels of c-MYC and CDKN2B proteins in frozen CTVT tissue lysates. In Figure-4a, Western blot analysis showed the expected c-MYC protein band at 49−51 kDa and the CDKN2B protein band at 15 kDa, with β-actin (42 kDa) as the loading control. c-MYC protein expression was higher in GTVT (1.29 ± 0.26-fold) than in ETVT (1.11 ± 0.45-fold), but this difference was not statistically significant (p = 0.72, Figure-4b). Similar to c-MYC, CDKN2B protein expression was lower in ETVT (0.11 ± 0.01-fold) than in GTVT (0.21 ± 0.017-fold), with a statistically significant difference between the two groups (p = 0.0069, Figure-4b). The results of the Western blotting were consistent with the results of the gene promoter methylation, gene expression, and IHC analyses in GTVTs and ETVTs, similar to those reported earlier in another section of this study.

## Discussion

With their unique characteristics, CTVTs offer a valuable model for studying human cancer, as exemplified by CTVTs [4]. This study is the first to provide a combined analysis of methylation, gene expression, and protein expression for c-MYC and *CDKN2B* in canine TVTs, offering a more holistic understanding of these critical genes in CTVTs. We have demonstrated that canine TVTs can harbor hypermethylated promoter genes in oncogenic and TSGs. Cancer-related promoter DNA methylation and gene expression vary across cancer types. Previous studies have reported that DNA methylation epigenetically silences the *MYC* gene in canine mammary tumors and the *NKX3.1* gene in prostate cancer. Interestingly, this silencing is similar in cancerous and healthy tissues [20, 23]. Conversely, next-generation sequencing analyses have revealed significant hypermethylation of CGIs within promoter regions across hundreds of genes in malignant melanoma compared to normal canine oral mucosa [24].

Under normal circumstances, most CpG sites are methylated, whereas most CGIs are unmethylated [15]. These CGIs localize near gene promoters and other gene-regulatory elements and tend to be hypomethylated [25]. In contrast, cancer cells exhibit widespread gene hypomethylation and CGI promoter hypermethylation. In this study, we revealed widespread hypermethylation of oncogenic genes (c-MYC) and TSGs (*CDKN2B*) in both genital and extragenital canine TVTs. Our results suggest that promoter DNA methylation is a potentially significant epigenetic factor in gene and protein regulation in CTVTs [11, 19]. This finding is further supported by the established roles of other epigenetic and genetic alterations, including histone modifications, 5-methylcytosine (m^5^C) RNA modification, non-coding RNAs (ncRNAs) and microRNAs, gene mutations, and copy number variations, in tumorigenesis [15, 26, 27].

In genital TVTs, DNA methylation of the c-MYC promoter region was higher than that in ETVTs. Real-time PCR, IHC, and Western blotting collectively revealed a significant downregulation of c-MYC expression in GTVTs, suggesting its potential role in the development or progression of these tumors. However, mutations, long ncRNAs, and LINE-1 sequence insertions in the canine c-MYC promoter have also been reported in CTVT, suggesting that these factors may also play a role in tumor senescence and progression [1, 9−11, 19, 28]. Therefore, we cannot definitively conclude that c-MYC hypermethylation was solely responsible for gene silencing because we did not perform a mutation analysis of that region in the current study.

MYC is a known repressor of genes involved in cell cycle regulation, particularly cell cycle inhibitors and growth arrest genes. Our findings demonstrate a lower methylation level in the MYC promoter of ETVTs, which hypothetically translates to increased MYC gene expression and protein production. MYC can typically suppress cell cycle inhibitor genes, such as cyclin-dependent kinase (CDK), by interacting with other proteins such as transcription factors (MYC-interacting zinc-finger protein 1 and specificity protein 1) and histone deacetylase 3 [29]. However, our data revealed an unexpected observation: Despite lower MYC promoter methylation in ETVTs compared to GTVTs, protein levels appeared similar in both tissue types as evidenced by IHC and Western blotting. This suggests the presence of additional regulatory mechanisms controlling MYC protein expression, which warrant further investigation.

DNA methylation patterns can distinguish between normal, tumor, and metastatic tumors [30]. Canine TVT can metastasize to extragenital tissues, as evidenced by the presence of circulating neoplastic cells in immunosuppressed dogs and the occurrence of metastases in the oral and nasal cavities, conjunctiva, eye, skin, and subcutis [3, 31, 32]. CTVT downregulates dog leukocyte antigen (DLA) class I and II genes (dog equivalents of major histocompatibility complex (MHC)-I and -II), hindering immune system recognition of tumor antigens on MHC I and promoting immune escape [9, 16, 33]. The observed downregulation of MHC genes may be caused by mutations in DLA genes or epigenetic mechanisms, mirroring the process in DFT1 tumors [8, 34]. Hypermethylation of genes involved in tumor progression and dissemination is a significant mechanism of tumorigenesis, particularly in metastatic tumors [15, 35]. Our findings, which showed that the methylation status of the c-MYC promoter region in ETVTs may be different, support this notion. Oncogenic c-MYC accelerates the cell cycle not only through CDK activity but also by downregulating a group of proteins that act as cell cycle checkpoints, including INK4A/p16 *(CDKN2A)*, p15 (*CDKN2B*), and p21 (*CDKN1A*) [35, 36]. The expression of CDKN2B, a protein crucial for cell cycle control, is partially inhibited by MYC in collaboration with antisense RNAs and protein products from *BMI1, TBX2*, *and P53 genes* [9, 20, 37]. Our study uncovered a perplexing phenomenon in *CDKN2B* gene and protein expression. While ETVTs had a much higher percentage *of CDKN2B* methylation than GTVTs, their gene expression was significantly higher. This suggests that regulatory mechanisms governing gene expression in these tumors go beyond simple methylation and warrant further investigation. While GTVTs displayed a clear link between methylation, gene, and protein levels, this correlation was absent in ETVTs. These observations suggest the existence of additional regulatory mechanisms beyond promoter methylation. These mechanisms may involve interactions with other protein-coding genes or transcriptional downregulation of ncRNAs, such as microRNAs: miR34a, miR-23b/27, miR17/92, as previously discussed [20, 35]. In addition, our report tested the methylation status of CDKN2B in the 5′ region of its CGI. However, this gene is minimal in dogs and has a small proportion of CGIs. The methylation status of a gene may vary across its CGI; therefore, it is vital to consider the size of the CGI when verifying gene hypermethylation. Even if hypermethylation of the gene promoter occurs at the gene level, we found that it does not influence transcriptional genes or the synthesis of corresponding proteins. In our report, the location of the tested methylation status of the *CDKN2B* gene is aligned with the 5′ region of the CGI, but the fact that this gene in canines is so small with a small proportion of CGI. The representation results may not reflect this gene’s actual hypermethylation status if we tested other regions within the gene. Although the precise information in this study is yet unknown, methylation at particular intragenic locations or other DNA areas, such as CGI coastlines or inside exon toward the 3′ end, may have more effects on transcriptional suppression than promoter methylation in CTVT [2]. Therefore, we suggest that DNA methylation plays a limited role in regulating these gene categories in CTVTs.

## Conclusion

Although most CTVT research has focused on genomic abnormalities, non-genomic mutation studies are emerging as alternative cancer detection and treatment methods. DNA methylation may play a crucial role in CTVT progression and control. Our findings indicate a significant methylation level in the oncogenes and TSGs MYC and *CDKN2B*. Additional research be conducted to determine how DNA methylation cooperates with DNA mutation in these cancers.

## Authors’ Contributions

SK: Investigation, formal analysis, visualization, and writing – original draft. KP and PP: Investigation and formal analysis. CS: Resources and supervision. ST: Resources and supervision. PC: Conceptualization, methodology, validation, formal analysis, data curation, visualization, supervision, project administration, funding acquisition, and writing review and editing. All authors have read, reviewed, and approved the final manuscript.
